# Association between urinary metallothionein concentration and causes of death among cadmium-exposed residents in Japan: a 35-year follow-up study

**DOI:** 10.1265/ehpm.24-00176

**Published:** 2025-01-07

**Authors:** Lianen Li, Rie Okamoto, Xian Liang Sun, Teruhiko Kido, Kazuhiro Nogawa, Yasushi Suwazono, Hideaki Nakagawa, Masaru Sakurai

**Affiliations:** 1 School of Medicine, and The First Affiliated Hospital, Huzhou University, 759 2nd Ring East Road, Huzhou 313000, China; 2 Faculty of Health Sciences, Institute of Medical Pharmaceutical and Health Sciences, Kanazawa University, 5-11-80 Kodatsuno, Kanazawa, Ishikawa, Japan; 3 Department of Occupational and Environmental Medicine, Graduate School of Medicine, Chiba University, 1-8-1 Inohana, Chuoku, Chiba, Japan; 4 Department of Social and Environmental Medicine, Kanazawa Medical University, 1-1 Daigaku, Uchinada, Ishikawa, Japan

**Keywords:** Urinary metallothionein, Cadmium, Cardiovascular disease, Tumor, Causes of death, Mortality

## Abstract

**Background:**

As research progresses, there is a growing body of evidence indicating that urinary metallothionein (MT) levels may be elevated in individuals exposed to cadmium (Cd). This study aimed to investigate the potential association between urinary MT levels and causes of mortality among residents of the Kakehashi River Basin who have been exposed to Cd.

**Method:**

The study involved a total of 1,398 men and 1,731 women were conducted between 1981 and 1982, with follow-up until November 2016. The study employed the Cox proportional-hazards model to examine the association between higher urinary MT concentrations and the risk of all-cause or cause-specific mortality within the population. Furthermore, the Fine and Gray competing risks regression model was used to evaluate the links between specific causes of death.

**Results:**

The findings revealed that elevated urinary MT concentrations were linked to increased all-cause mortality and higher mortality rates from renal and urinary tract diseases across all participants. Specifically, in men, higher urinary MT levels were associated with elevated all-cause mortality, while in women, increased concentrations were linked to higher mortality from endocrine, nutritional, and metabolic diseases, as well as cardiovascular diseases. Even after adjusting for competing risks, higher urinary MT concentrations were associated with tumor-related mortality in men and continued to be associated with cardiovascular disease mortality in women.

**Conclusions:**

In conclusion, the results suggest that women may face a greater risk of adverse health effects due to prolonged exposure to Cd. Urinary MT levels could potentially serve as a biomarker for mortality from these diseases in populations chronically exposed to Cd.

**Supplementary information:**

The online version contains supplementary material available at https://doi.org/10.1265/ehpm.24-00176.

## Graphical Abstract

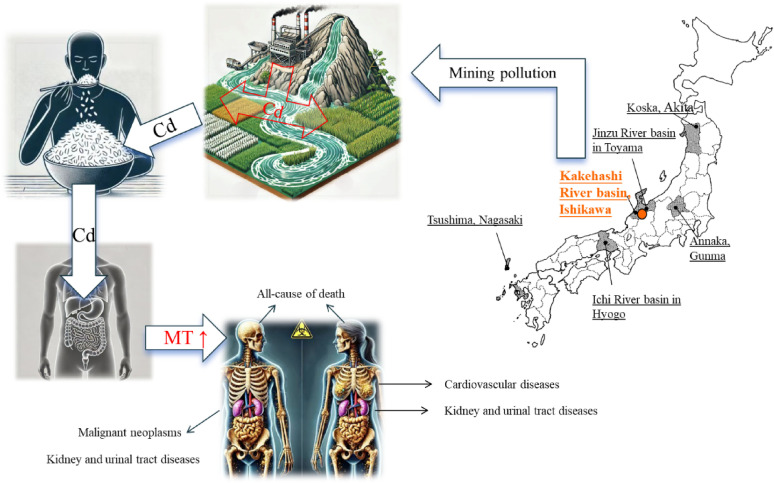

## 1. Background

The Cd pollution in Ishikawa Prefecture’s Kakehashi River basin has been a serious issue that has affected the region for a long time [1]. The pollution can be traced back to extensive mining activities that started in the 17th century in the upper parts of the river and continued until the government stopped mining in 1971. To resolve the pollution problem, the government implemented soil treatment and replacement efforts between 1977 and 1988 to restore the contaminated rice fields [2].

Cd occurs naturally in tiny quantities, the rise in environmental Cd levels is primarily due to human activities, including industrial processes, pollution, waste incineration, and e-waste recycling. These activities result in Cd becoming widespread in the air, water, and soil, and ultimately entering the human body via ingestion and inhalation [3]. Cd accumulates in tissues such as the liver and kidneys, it has a biological half-life of 10–35 years in the human body [4]. Prolonged occupational and non-occupational exposure to Cd can increase the risk of death from diseases such as cancer [5, 6], kidney disease [7–9], and cardiovascular disease [10–12].

MT can be induced in the liver by various factors like metals (e.g., Cd), drugs, and inflammatory mediators [13]. Due to its rich sulfhydryl content and high affinity for divalent heavy metal ions, MT can bind with many heavy metals such as Cd and mercury to protect cells and tissues from heavy metal toxicity [14]. The metal-thiolate fractions of MT are dynamic and have a high affinity for metals, which can promote metal exchange in tissues [15]. This property makes MT and Cd combine to form a complex and play an important role in the transfer process of plasma to the kidney, finally entering the primary urine through the glomerular membrane [16]. Urinary MT excretion is associated not only with Cd exposure but also with several other proteins that are used as indicators of renal tubule dysfunction, such as β_2_-microglobulin (β_2_-MG) and retinol-binding proteins [17–19].

Existing studies on Cd-exposed inhabitants in this area mainly focus on the adverse effects of Cd intake [1, 2, 19], urinary Cd [20], and urinary β_2_-MG [21] on human health, few studies seem to have found a relationship between urinary MT concentration and all-cause and specific cause mortality. We conducted this study to investigate the relationship between urinary MT concentrations and causes of death in residents of Cd-exposed areas and whether urinary MT can be used as a marker of adverse prognosis due to Cd exposure.

## 2. Materials and methods

### 2.1 Study population

The study included 3,178 residents (1,424 men and 1,754 women) of the Cd-exposed Kakehashi River basin who participated in the Ishikawa Prefecture Health Effects Survey conducted in 1981–1982. The population was studied for residents aged 50 and older who lived in the area at the time. Urine samples are frozen and stored at −20 °C within 6 hours of collection, with 4 ml of equal samples per specimen. Subsequently, the frozen specimens are shipped to the United States (where the samples are analyzed), packaged in dry ice, and kept at −85 °C until analysis [22]. The urinary MT concentration was determined by single antibody radioimmunoassay [23]. Urinary creatinine (Cr) was analyzed according to the method of Bonsnes and Taussky [24].

### 2.2 Follow-up

Participants were prospectively followed up from the day of the initial examination during the 1981–1982 Health Effects Survey through November 2016. The survival status of the participants was confirmed in 1991, 1998, 2003, and 2016. This study analyzed 35 years of follow-up data. The causes of death are classified according to the International Statistical Classification of Diseases and Related Health Problems, the 9th revision before 1994 (ICD-9) and ICD-10 after 1995. Of the 3178 potential participants, 49 were excluded for the following reasons: urinary MT concentration data were missing (n = 10), cause of death could not be determined (n = 39). 3,129 people were eventually included in this study.

### 2.3 Statistical analysis

After adjusting for age, the Cox proportional-hazards model was used to analyze death risk in the general population and different genders. This regression analysis model is used to investigate associations between survival time and relevant factors from the baseline survey date to the subject endpoint. Urinary MT concentration (µg/g Cr) (≤116; References, >116–197, >197–342, and >342) assessed risk ratios (HRs) for all-cause and cause-specific death. In addition, age-adjusted HRs were calculated when urinary MT concentration increased 300 µg/g Cr. These analyses were performed using the Social Science Statistical Package (SPSS^®^, version 19; IBM Corporation, Armonk, New York, USA). The Fine and Gray competing risks regression model was carried out using the cmprsk package (Version: 2.2-11) of R statistical software (version 4.3.3; R Core Team, 2024; R Foundation for Statistical Computing, Vienna, Austria), and age-adjusted relative risks (RRs) for cause-specific death were calculated for 300-µg/g Cr increases in the MT concentration in which death from other causes was included in the model as competing risks. Most mammalian tissues contain age-related basal levels of MT because MT is involved in processes including cell growth and proliferation and intracellular metal regulation [15]. So, we adjusted for age when assessing the association between urinary MT concentration and risk of death.

## 3. Results

Table 1 shows the number of participants, mortality (mortality rate), mean follow-up duration per person (years), and the geometric mean of MT concentration, based on gender classification. In this study, the mean age (standard deviation) of men was 63.30 (9.11) years, and 63.86 (9.28) years for women. Among them, 1,104 male participants died during our observation period, accounting for 79.0% of male participants; 1,114 female participants died, accounting for 64.4% of female participants. Among the male participants, the geometrical mean of urinary MT concentration was 157.63 µg/g Cr and 248.04 µg/g Cr for female participants.

**Table 1:** Number of participants, person-years of follow-up and geometrical mean of urinary biochemical index, metallothionein.

**Characteristics**	**Male**	**Female**
Number of participants	1398	1731
Number of mortalities during the follow-up period (%)	1104 (79.0)	1114 (64.4)
Mean age (years)	63.3	63.86
Mean follow-up duration per person (years)	18.17	20.84
Urinary mean metallothionein (µg/g Cr) & (GSD)	157.63 (2.24)	248.04 (2.16)

GSD: geometric standard deviation

Table 2 shows the survival and death of participants of different genders, age brackets, and concentrations of urinary MT. In the urinary MT concentration corresponding to each age bracket of male and female participants. In all three subgroups of participants over the age of 60, both male and female, there were more deaths than survivors by the end of our observation period.

**Table 2:** The mortality of participants according to urinary metallothionein concentration, age bracket and gender.

**Characteristics**	**Urinary MT concentration (µg/g Cr)**							

	**≤116**		**>116–197**		**>197–342**		**>342**	
	
	**S (%)**	**D (%)**	**S (%)**	**D (%)**	**S (%)**	**D (%)**	**S (%)**	**D (%)**
Male	N = 149	N = 373	N = 63	N = 307	N = 48	N = 221	N = 34	N = 203
50–59	122 (81.9)	156 (41.8)	52 (82.5)	114 (37.1)	35 (72.9)	58 (26.2)	20 (58.8)	38 (18.7)
60–69	21 (14.1)	123 (33.0)	8 (12.7)	107 (34.9)	12 (25.0)	93 (42.1)	13 (38.2)	97 (47.8)
70–79	5 (3.4)	78 (20.9)	3 (4.8)	72 (23.5)	1 (2.1)	56 (25.3)	0 (0)	49 (24.1)
80+	1 (0.7)	16 (4.3)	0 (0)	14 (4.6)	0 (0)	14 (6.3)	1 (2.9)	19 (9.4)

Female	N = 119	N = 141	N = 176	N = 237	N = 171	N = 342	N = 151	N = 394
50–59	84 (70.6)	45 (31.9)	139 (79.0)	65 (27.4)	132 (77.2)	79 (23.1)	93 (61.6)	63 (16.0)
60–69	29 (24.4)	48 (34.0)	30 (17.0)	91 (38.4)	35 (20.5)	142 (41.5)	48 (31.8)	162 (41.1)
70–79	6 (5.0)	41 (29.1)	5 (2.8)	61 (25.7)	4 (2.3)	92 (26.9)	10 (6.6)	119 (30.2)
80+	0 (0)	7 (5.0)	2 (1.1)	20 (8.4)	0 (0)	29 (8.5)	0 (0)	50 (12.7)

MT: metallothionein, N: number of participants, S: number of survivals, D: number of deaths.

Table 3 shows the cause of death and mortality of different genders grouped according to the concentration of urinary MT. Among male participants, the most common cause of death in urinary MT concentration of ≤116 µg/g Cr was Malignant neoplasms; for participants with urinary MT concentration above 116 µg/g Cr, the most common cause of death was circulatory diseases. Among female participants, the most common cause of death was circulatory disease.

**Table 3:** Causes of death of the participants according to gender and the concentration of metallothionein.

**Characteristics**	**Male, D (%)**				**Female, D (%)**			
**MT concentration (µg/g Cr)**	**≤116 (N = 522)**	**>116–197 (N = 370)**	**>197–342 (N = 269)**	**>342 (N = 237)**	**≤116 (N = 260)**	**>116–197 (N = 413)**	**>197–342 (N = 513)**	**>342 (N = 545)**
All-cause of death	373 (71.5)	307 (83.0)	221 (82.2)	203 (85.7)	141 (54.2)	237 (57.4)	342 (66.7)	394 (72.3)
Cause-specific death								
Malignant neoplasms	117 (22.4)	90 (24.3)	60 (22.3)	44 (18.6)	35 (13.5)	53 (12.8)	71 (13.8)	73 (13.4)
Oesophagus	3 (0.6)	2 (0.5)	0	0	0	0	1 (0.2)	3 (0.6)
Stomach	25 (4.8)	17 (4.6)	8 (3.0)	6 (2.5)	4 (1.5)	7 (1.7)	11 (2.1)	10 (1.8)
Colon rectum	14 (2.7)	9 (2.4)	7 (2.6)	5 (2.1)	6 (2.3)	10 (2.4)	8 (1.6)	11 (2.0)
Liver	17 (3.3)	11 (3.0)	4 (1.5)	2 (0.8)	4 (1.5)	1 (0.2)	8 (1.6)	5 (0.9)
Pancreas	10 (1.9)	6 (1.6)	8 (3.0)	6 (2.5)	5 (1.9)	10 (2.4)	8 (1.6)	10 (1.8)
Lung	23 (4.4)	23 (6.2)	17 (6.3)	11 (4.6)	4 (1.5)	9 (2.2)	6 (1.2)	10 (1.8)
Prostate	7 (1.3)	3 (0.8)	4 (1.5)	3 (1.3)	0	0	0	0
Breast	0	0	0	0	3 (1.2)	2 (0.5)	5 (1.0)	1 (0.2)
Uterus	0	0	0	0	0	0	2 (0.4)	0
Ovary	0	0	0	0	0	2 (0.5)	3 (0.6)	2 (0.4)
Bladder	2 (0.4)	5 (1.4)	0	1 (0.4)	0	1 (0.2)	1 (0.2)	3 (0.6)
Kidney	1 (0.2)	3 (0.8)	0	0	0	0	0	1 (0.2)
Lymphatic or hematopoietic tissue	7 (1.3)	3 (0.8)	3 (1.1)	3 (1.3)	2 (0.8)	5 (1.2)	3 (0.6)	7 (1.3)
Endocrine, nutritional, and metabolic diseases	6 (1.1)	6 (1.6)	1 (0.4)	2 (0.8)	1 (0.4)	4 (1.0)	14 (2.7)	6 (1.1)
Diabetes	5 (1.0)	3 (0.8)	0	2 (0.8)	1 (0.4)	3 (0.7)	8 (1.6)	4 (0.7)
Circulatory diseases	101 (19.3)	92 (24.9)	70 (26.0)	62 (26.2)	43 (16.5)	84 (20.3)	123 (24.0)	142 (26.1)
Cardiovascular diseases	49 (9.4)	49 (13.2)	35 (13.0)	27 (11.4)	19 (7.3)	49 (11.9)	72 (14.0)	67 (12.3)
Ischemic Heart diseases	11 (2.1)	18 (4.9)	8 (3.0)	10 (4.2)	3 (1.2)	8 (1.9)	16 (3.1)	19 (3.5)
Heart failure	25 (4.8)	27 (7.3)	19 (7.1)	12 (5.1)	10 (3.8)	33 (8.0)	47 (9.2)	35 (6.4)
Other heart diseases	13 (2.5)	4 (1.1)	8 (3.0)	5 (2.1)	6 (2.3)	8 (1.9)	9 (1.8)	13 (2.4)
Cerebrovascular diseases	44 (8.4)	41 (11.1)	32 (11.9)	29 (12.2)	23 (8.8)	32 (7.7)	50 (9.7)	71 (13.0)
Cerebral hemorrhage	9 (1.7)	5 (1.4)	11 (4.1)	1 (0.4)	4 (1.5)	6 (1.5)	11 (2.1)	14 (2.6)
Cerebral infarction	22 (4.2)	19 (5.1)	9 (3.3)	15 (6.3)	11 (4.2)	22 (5.3)	22 (4.3)	16 (2.9)
Subarachnoid hemorrhage	1 (0.2)	0	3 (1.1)	0	1 (0.4)	1 (0.2)	5 (1.0)	9 (1.7)
Other cerebrovascular diseases	12 (2.3)	17 (4.6)	9 (3.3)	13 (5.5)	7 (2.7)	3 (0.7)	12 (2.3)	32 (5.9)
Diseases of the respiratory systems	66 (12.6)	44 (11.9)	39 (14.5)	39 (16.5)	23 (8.8)	29 (7.0)	43 (8.4)	44 (8.1)
Pneumonia and influenza	40 (7.7)	25 (6.8)	23 (8.6)	20 (8.4)	14 (5.4)	22 (5.3)	30 (5.8)	30 (5.5)
Diseases of the digestive systems	17 (3.3)	8 (2.2)	9 (3.3)	12 (5.1)	4 (1.5)	10 (2.4)	15 (2.9)	22 (4.0)
Gastric and duodenal ulcer	2 (0.4)	0	1 (0.4)	2 (0.8)	0	2 (0.5)	0	1 (0.2)
Ileus and intestinal obstruction	3 (0.6)	2 (0.5)	1 (0.4)	0	0	2 (0.5)	2 (0.4)	3 (0.6)
Liver cirrhosis	4 (0.8)	1 (0.3)	1 (0.4)	9 (3.8)	0	2 (0.5)	3 (0.6)	7 (1.3)
Kidney and urinal tract diseases	8 (1.5)	12 (3.2)	8 (3.0)	10 (4.2)	2 (0.8)	5 (1.2)	12 (2.3)	18 (3.3)
Renal diseases	0	1 (0.3)	0	1 (0.4)	0	0	3 (0.6)	2 (0.4)
Renal failure	7 (1.3)	8 (2.2)	4 (1.5)	8 (3.4)	2 (0.8)	3 (0.7)	6 (1.2)	13 (2.4)
Other renal diseases	0	0	1 (0.4)	0	0	2 (0.5)	2 (0.4)	2 (0.4)
Senility	19 (3.6)	18 (4.9)	6 (2.2)	11 (4.6)	13 (5.0)	28 (6.8)	30 (5.8)	47 (8.6)
External causes of mortality	19 (3.6)	12 (3.2)	15 (5.6)	12 (5.1)	6 (2.3)	10 (2.4)	11 (2.1)	19 (3.5)
Toxic effects	1 (0.2)	0	0	0	0	1 (0.2)	1 (0.2)	0

MT: metallothionein, D: number of deaths.

Table 4 shows the Hazard Ratios (HRs) for all-cause and cause-specific mortality among residents based on their urinary MT concentrations. No statistically significant differences were found in all causes of death across the groups. However, significant differences were observed for cause-specific deaths. For cardiovascular diseases, the HRs (95% CI) were 1.38 (1.01–1.88) for participants with a urinary MT concentration of >116–197 µg/g Cr and 1.41 (1.04–1.91) for participants with a urinary MT concentration of >197–342 µg/g Cr compared to those with a concentration of ≤116 µg/g Cr. In the case of kidney and urinary tract diseases, the HR (95% CI) for participants with a urinary MT concentration of >342 µg/g Cr was 2.19 (1.06–4.52) compared to those with a concentration of ≤116 µg/g Cr. Conversely, for malignant tumors, the HR (95% CI) was 0.76 (0.59–0.97) for participants with a urinary MT concentration of >342 µg/g Cr compared to those with a concentration of ≤116 µg/g Cr. Moreover, the HR for a 300 µg/g Cr increase in urinary MT was lower for malignant neoplasms, indicating a linear association between MT concentration and mortality risk for malignant neoplasms.

**Table 4:** Age-adjusted risk of all-cause and cause-specific mortality according to urinary metallothionein concentrations in all residents.

**Characteristics**	**Urinary MT concentration (µg/g Cr)**											**MT (µg/g Cr)**	

	**≤116 (N = 782)**		**>116–197 (N = 783)**			**>197–342 (N = 782)**			**>342 (N = 782)**			**+300(continuous)**	
**Cause of death**	**D**	**HR (95% CI)**	**D**	**HR (95% CI)**	**P**	**D**	**HR (95% CI)**	**P**	**D**	**HR (95% CI)**	**P**	**HR (95% CI)**	**P**
All-cause of death	514	1.00 (reference)	544	1.02 (0.90–1.15)	0.79	563	1.00 (0.89–1.13)	1.00	597	0.98 (0.87–1.10)	0.98	0.95 (0.87–1.04)	0.29
Cause-specific death													
Malignant neoplasms	152	1.00 (reference)	143	0.92 (0.73–1.16)	0.48	131	0.83 (0.66–1.05)	0.12	117	**0.76 (0.59–0.9**7)	**0.02**	**0.79 ** **(0.65–0.96)**	**0.02**
Circulatory diseases	144	1.00 (reference)	176	1.16 (0.93–1.45)	0.18	193	1.20 (0.97–1.49)	0.10	204	1.11 (0.90–1.38)	0.33	1.00 (0.86–1.17)	1.00
Cardiovascular diseases	68	1.00 (reference)	98	**1.38 (1.01–1.88)**	**0.04**	107	**1.41 (1.04–1.91)**	**0.03**	94	1.07 (0.79–1.47)	0.65	0.86 (0.69–1.08)	0.20
Cerebrovascular diseases	67	1.00 (reference)	73	1.04 (0.75–1.45)	0.83	82	1.10 (0.80–1.52)	0.57	100	1.19 (0.87–1.63)	0.27	1.16 (0.92–1.45)	0.22
Diseases of the respiratory systems	89	1.00 (reference)	73	0.79 (0.58–1.08)	0.14	82	0.84 (0.62–1.13)	0.25	83	0.78 (0.58–1.06)	0.11	0.92 (0.73–1.17)	0.49
Diseases of the digestive systems	21	1.00 (reference)	18	0.82 (0.44–1.54)	0.54	24	1.05 (0.58–1.88)	0.88	34	1.37 (0.79–2.37)	0.26	1.43 (0.95–2.15)	0.09
Kidney and urinal tract diseases	10	1.00 (reference)	17	1.64 (0.75–3.59)	0.21	20	1.80 (0.84–3.85)	0.13	28	**2.19 (1.06–4.52)**	**0.04**	1.55 (0.98–2.46)	0.06
Senility	32	1.00 (reference)	46	1.32 (0.84–2.08)	0.23	36	0.99 (0.61–1.59)	0.95	58	1.05 (0.68–1.62)	0.84	0.86 (0.63–1.18)	0.86
External causes of mortality	25	1.00 (reference)	22	0.87 (0.49–1.54)	0.63	26	1.02 (0.59–1.77)	0.95	31	1.25 (0.73–2.12)	0.42	1.12 (0.74–1.70)	0.58

MT: metallothionein, N: number of participants, D: number of deaths, P: *p*-value, HR: hazard ratio, CI: confidence interval. Bold figures show significant HR.

Table 5 shows the HRs of all-cause mortality and cause-specific mortality in groups of male participants according to urinary MT concentrations. We found that the risk of all-cause mortality, HRs for groups of urinary MT concentration >116–197 µg/g Cr (HR: 1.18, 95% CI: 1.01–1.37) and >342 µg/g Cr (HR: 1.20, 95% CI: 1.01–1.43) were higher compared with groups of urinary MT concentration ≤116 µg/g Cr.

**Table 5:** Age-adjusted risk of all-cause and cause-specific mortality according to urinary metallothionein concentrations in male residents.

**Characteristics**	**Urinary MT concentration (µg/g Cr)**											**MT (µg/g Cr)**	

	**≤116 (N = 522)**		**>116–197 (N = 370)**			**>197–342 (N = 269)**			**>342 (N = 237)**			**+300(continuous)**	
**Cause of death**	**D**	**HR (95% CI)**	**D**	**HR (95% CI)**	**P**	**D**	**HR (95% CI)**	**P**	**D**	**HR (95% CI)**	**P**	**HR (95% CI)**	**P**
All-cause of death	373	1.00 (reference)	307	**1.18 (1.01–1.37)**	**0.03**	221	1.16 (0.98–1.37)	0.08	203	**1.20 (1.01–1.43)**	**0.04**	1.12 (0.97–1.29)	0.14
Cause-specific death													
Malignant neoplasms	117	1.00 (reference)	90	1.13 (0.86–1.49)	0.38	60	1.07 (0.78–1.47)	0.67	44	0.94 (0.66–1.34)	0.74	0.95 (0.70–1.26)	0.72
Circulatory diseases	101	1.00 (reference)	92	1.28 (0.96–1.69)	0.09	70	1.31 (0.96–1.77)	0.09	62	1.22 (0.88–1.68)	0.23	1.19 (0.92–1.54)	0.19
Cardiovascular diseases	49	1.00 (reference)	49	1.41 (0.95–2.09)	0.09	35	1.34 (0.86–2.07)	0.19	27	1.08 (0.67–1.73)	0.76	1.02 (0.70–1.50)	0.92
Cerebrovascular diseases	44	1.00 (reference)	41	1.30 (0.85–1.99)	0.23	32	1.38 (0.87–2.17)	0.17	29	1.33 (0.83–2.14)	0.24	1.27 (0.87–1.85)	0.22
Diseases of the respiratory systems	66	1.00 (reference)	44	0.98 (0.67–1.43)	0.90	39	1.18 (0.79–1.75)	0.42	39	1.33 (0.89–1.98)	0.17	1.23 (0.87–1.74)	0.23
Diseases of the digestive systems	17	1.00 (reference)	8	0.68 (0.29–1.58)	0.37	9	1.08 (0.48–2.43)	0.86	12	1.67 (0.78–3.55)	0.18	1.69 (0.88–3.25)	0.11
Kidney and urinal tract diseases	8	1.00 (reference)	12	2.18 (0.89–5.34)	0.09	8	1.88 (0.70–5.03)	0.21	10	2.38 (0.92–6.11)	0.07	1.50 (0.73–3.05)	0.27
Senility	19	1.00 (reference)	18	1.21 (0.63–2.32)	0.57	6	0.53 (0.21–1.34)	0.18	11	0.74 (0.34–1.58)	0.43	0.71 (0.36–1.39)	0.32
External causes of mortality	19	1.00 (reference)	12	0.92 (0.44–1.89)	0.81	15	1.64 (0.83–3.24)	0.16	12	1.60 (0.77–3.33)	0.21	1.31 (0.70–2.44)	0.40

MT: metallothionein, N: number of participants, D: number of deaths, P: p-value, HR: hazard ratio, CI: confidence interval. Bold figures show significant HR.

Table 6 shows HRs for all-cause mortality and cause-specific mortality among female participants based on their urinary MT concentrations. In terms of all-cause mortality, the HRs (95% CI) were 1.30 (1.07–1.59) for participants with a urinary MT concentration of >197–342 µg/g Cr and 1.28 (1.05–1.55) for those with a concentration of >342 µg/g Cr, as compared to those with ≤116 µg/g Cr. Additionally, the HR (95% CI) for circulatory diseases was 1.46 (1.03–2.05) in the >342 µg/g Cr group compared to the ≤116 µg/g Cr group. Regarding cardiovascular diseases, the HRs (95% CI) were 1.80 (1.06–3.05) and 2.09 (1.26–3.46) for participants with urinary MT concentrations of >116–197 µg/g Cr and >197–342 µg/g Cr, respectively, in comparison to those with ≤116 µg/g Cr.

**Table 6:** Age-adjusted risk of all-cause and cause-specific mortality according to urinary metallothionein concentrations in female residents.

**Characteristics**	**Urinary MT concentration (µg/g Cr)**											**MT (µg/g Cr)**	

	**≤116 (N = 260)**		**>116–197 (N = 413)**			**>197–342 (N = 513)**			**>342 (N = 545)**			**+300(continuous)**	
**Cause of death**	**D**	**HR (95% CI)**	**D**	**HR (95% CI)**	**P**	**D**	**HR (95% CI)**	**P**	**D**	**HR (95% CI)**	**P**	**HR (95% CI)**	**P**
All-cause of death	141	1.00 (reference)	237	1.14 (0.92–1.40)	0.23	342	**1.30 ** **(1.07–1.59)**	**0.01**	394	**1.28 ** **(1.05–1.55)**	**0.01**	1.08 (0.95–1.21)	0.24
Cause-specific death													
Malignant neoplasms	35	1.00 (reference)	53	0.98 (0.64–1.50)	0.92	71	1.08 (0.72–1.62)	0.72	73	1.07 (0.71–1.60)	0.74	0.97 (0.74–1.26)	0.79
Circulatory diseases	43	1.00 (reference)	84	1.33 (0.92–1.92)	0.13	123	**1.54 ** **(1.09–2.18)**	**0.02**	142	**1.46 ** **(1.03–2.05)**	**0.03**	1.06 (0.86–1.29)	0.60
Cardiovascular diseases	19	1.00 (reference)	49	**1.80 (1.06–3.05)**	**0.03**	72	**2.09 ** **(1.26–3.46)**	**<0.01**	67	1.58 (0.95–2.63)	0.08	0.90 (0.68–1.19)	0.44
Cerebrovascular diseases	23	1.00 (reference)	32	0.93 (0.55–1.59)	0.8	50	1.15 (0.70–1.88)	0.59	71	1.36 (0.85–2.18)	0.20	1.28 (0.95–1.73)	0.10
Diseases of the respiratory systems	23	1.00 (reference)	29	0.88 (0.51–1.53)	0.66	43	1.05 (0.63–1.74)	0.85	44	0.92 (0.55–1.52)	0.73	1.11 (0.79–1.54)	0.55
Diseases of the digestive systems	4	1.00 (reference)	10	1.73 (0.54–5.52)	0.35	15	2.03 (0.67–6.12)	0.21	22	2.42 (0.83–7.06)	0.10	1.56 (0.90–2.72)	0.12
Kidney and urinal tract diseases	2	1.00 (reference)	5	1.71 (0.33–8.82)	0.52	12	3.31 (0.74–14.83)	0.12	18	4.15 (0.96–17.93)	0.06	**2.35 ** **(1.20–4.59)**	**0.01**
Senility	13	1.00 (reference)	28	1.53 (0.79–2.96)	0.21	30	1.34 (0.69–2.59)	0.38	47	1.32 (0.71–2.46)	0.38	0.90 (0.63–1.31)	0.59
External causes of mortality	6	1.00 (reference)	10	1.07 (0.39–2.95)	0.89	11	0.98 (0.36–2.64)	0.96	19	1.68 (0.64–4.04)	0.31	1.36 (0.76–2.45)	0.30

MT: metallothionein, N: number of participants, D: number of deaths, P: p-value, HR: hazard ratio, CI: confidence interval. Bold figures show significant HR.

Tables S1–S3, which correspond to Tables 4–6 in this paper, present the calculated risk ratios per standard deviation for all, male, and female residents, respectively. For all residents (Table S1), the HR per standard deviation (95% CI) for mortality from malignant tumors was 0.68 (0.54–0.85). The HR per standard deviation (95% CI) for mortality from kidney disease was 1.72 (1.00–2.97). In men (Table S2), the HR per standard deviation (95% CI) for all-cause mortality was 1.21 (1.03–1.42). For women (Table S3), the HR per standard deviation (95% CI) for all-cause mortality was 1.22 (1.04–1.42), and for mortality from kidney and urinal tract diseases, it was 2.94 (1.38–6.23).

Table 7 displays the age-adjusted relative risks (RRs) for MT levels (300 µg/g Cr higher) for various causes of death, as calculated by the Fine and Gray competing risk regression model. Among men, elevated urinary MT concentrations were associated with increased risks of stomach cancer (RR: 1.22, 95% CI: 1.02–1.46), colorectal cancer (RR: 1.20, 95% CI: 1.03–1.41), liver cancer (RR: 1.22, 95% CI: 1.02–1.44), cerebral hemorrhage (RR: 1.21, 95% CI: 1.02–1.43), and pneumonia and influenza (RR: 1.21, 95% CI: 1.03–1.43). In women, higher urinary MT levels were linked to increased risks of death from cerebral infarction (RR: 1.16, 95% CI: 1.01–1.32) and cardiovascular diseases (RR: 1.20, 95% CI: 1.03–1.39), particularly heart failure (RR: 1.22, 95% CI: 1.05–1.41). Notably, the relationship between urinary MT levels and cardiovascular diseases was observed in both Cox proportional risk and Fine and Gray competing risk regression models.

**Table 7:** Age-adjusted risk ratio of every cause-specific mortality according to urinary metallothionein concentrations.

	**Male, aRR (95%CI)**		**Female, aRR (95%CI)**	
	**MT, µg/g Cr**		**MT, µg/g Cr**	
	**+300 (continuous)**	**P value**	**+300 (continuous)**	**P value**
Malignant neoplasms	1.21 (0.99–1.47)	0.06	1.04 (0.89–1.21)	0.65
Oesophagus	1.16 (0.99–1.36)	0.06	1.01 (0.88–1.15)	0.93
Stomach	1.22 (1.02–1.46)	0.03	1.04 (0.92–1.19)	0.51
Colon rectum	1.20 (1.03–1.41)	0.02	1.07 (0.95–1.22)	0.27
Liver	1.22 (1.02–1.44)	0.03	1.07 (0.95–1.21)	0.28
Pancreas	1.09 (0.93–1.28)	0.30	1.06 (0.92–1.21)	0.41
Lung	1.05 (0.85–1.29)	0.67	1.04 (0.92–1.19)	0.51
Prostate	1.11 (0.95–1.31)	0.20	NA	
Breast	NA		1.13 (0.99–1.28)	0.07
Uterus	NA		1.08 (0.96–1.22)	0.21
Ovary	NA		1.11 (0.97–1.26)	0.13
Bladder	1.08 (0.90–1.29)	0.42	1.04 (0.91–1.18)	0.55
Kidney	1.14 (0.98–1.34)	0.09	1.07 (0.94–1.20)	0.31
Lymphatic or hematopoietic tissue	1.10 (0.92–1.30)	0.29	1.06 (0.93–1.20)	0.38
Endocrine nutritional and metabolic diseases	1.12 (0.96–1.31)	0.15	1.06 (0.93–1.21)	0.37
Diabetes	1.11 (0.95–1.30)	0.19	1.09 (0.97–1.24)	0.16
Circulatory diseases	1.03 (0.85–1.25)	0.79	1.05 (0.90–1.23)	0.55
Cardiovascular diseases	1.07 (0.88–1.29)	0.51	1.20 (1.03–1.39)	0.02
Ischemic Heart diseases	1.11 (0.92–1.34)	0.26	1.09 (0.93–1.26)	0.28
Heart failure	1.10 (0.91–1.34)	0.32	1.22 (1.05–1.41)	0.01
Cerebrovascular diseases	1.07 (0.89–1.29)	0.45	0.90 (0.77–1.05)	0.17
Cerebral hemorrhage	1.21 (1.02–1.43)	0.03	1.06 (0.91–1.25)	0.45
Cerebral infarction	1.05 (0.88–1.25)	0.59	1.16 (1.01–1.32)	0.04
Subarachnoid hemorrhage	1.13 (0.97–1.33)	0.12	1.03 (0.91–1.16)	0.68
Diseases of the respiratory systems	1.07 (0.88–1.28)	0.50	1.03 (0.89–1.20)	0.65
Pneumonia and influenza	1.21 (1.03–1.43)	0.02	1.04 (0.89–1.21)	0.62
Diseases of the digestive systems	0.99 (0.81–1.21)	0.92	1.03 (0.89–1.19)	0.66
Gastric and duodenal ulcer	1.00 (0.82–1.24)	0.97	1.08 (0.95–1.22)	0.22
Ileus and intestinal obstruction	1.15 (0.99–1.35)	0.08	1.10 (0.96–1.25)	0.17
Liver cirrhosis	0.93 (0.77–1.11)	0.42	1.04 (0.92–1.17)	0.57
Kidney and urinal tract diseases	1.02 (0.83–1.24)	0.87	0.99 (0.86–1.13)	0.86
Renal diseases	1.09 (0.93–1.28)	0.27	1.06 (0.94–1.20)	0.35
Renal failure	1.02 (0.84–1.25)	0.82	0.98 (0.86–1.13)	0.82
Senility	1.12 (0.93–1.36)	0.23	1.10 (0.95–1.27)	0.19
External causes of mortality	1.04 (0.86–1.26)	0.67	1.11 (0.96–1.28)	0.15

aRR: age-adjusted risk ratio, MT: metallothionein, CI: confidence interval, P: p-value.

## 4. Discussion

Our research has found an association between higher levels of urinary MT concentrations and an increased risk of kidney and urinary tract disease mortality, especially among women with a continuous increase in urinary MT concentration of 300 µg/g Cr. Previous studies have also observed that individuals exposed to Cd in the Kakehashi River Basin [7, 9] and the Jinzu River Basin [25] are more likely to experience kidney diseases. While studies using urinary β_2_-MG as an indicator have shown clear results in studying the relationship with renal disease mortality, the relationship between urinary MT and renal disease mortality is not as prominent. This could be due to urinary MT being more specific in reflecting renal impairment than urinary β_2_-MG, albeit less sensitive [24]. The kidney, being a crucial organ, can be significantly affected by prolonged exposure to Cd [4]. Furthermore, an increase in urinary MT excretion may indicate excessive Cd exposure and renal dysfunction resulting from long-term exposure to this element [26]. These findings suggest that chronic Cd exposure may elevate the risk of developing kidney and urinary tract diseases in individuals residing in contaminated areas.

We utilized the Cox proportional-hazards model to reveal an association between elevated levels of urinary MT and lower rates of mortality from malignant tumors in all residents who lived in the Kakehashi River Basin. This association demonstrated a linear relationship, which may be due to the pivotal role that MT plays in various biological processes, including metal binding, homeostasis, detoxification, cell growth, and proliferation regulation, as well as protection against DNA damage and oxidative stress [15]. However, when analyzing using the Fine and Gray competing risks regression model, we observed that higher levels of urinary MT in male participants resulted in higher mortality rates from stomach, colon, and liver cancers. Previous studies have explained the role of MT 2A in tumor progression in gastric cancer [27] and colorectal cancer [28, 29]. That’s consistent with what we’ve observed. Furthermore, our research revealed an association between elevated urinary MT levels and increased mortality rates from respiratory diseases in men according to the Fine and Gray competing risks regression model. In a population-longitudinal cohort study by Lee et al. [30], it was found that exposure to Cd from air pollution was associated with increased incidence and mortality of lung cancer. These suggest that the increased incidence and mortality of cancer caused by Cd may be related to the route of exposure.

In female participants, higher levels of urinary MT were associated with an increased risk of mortality from circulatory diseases, especially cardiovascular diseases. This connection was identified using the Cox proportional-hazards model and Fine and Gray competing risks regression mode. In a study conducted by Lin et al. [31], it was observed that exposure to cadmium altered cardiac lipid metabolism, leading to an increase in pro-inflammatory lipids and affecting inflammatory cytokine secretion, ultimately leading to heart inflammation in mice. In a vitro study by Chen et al. [32], it was demonstrated that Cd leads to the apoptosis of human umbilical vein endothelial cells and induces the production of inflammatory cytokines. Similarly, Fittipaldi et al. [33] discovered that Cd can disrupt the level and function of sex steroid hormone receptors, thereby impacting intracellular signaling associated with the pro-inflammatory state of endothelial cells, consequently resulting in endothelial cell impairment and vascular dysfunction. A 22-year follow-up study of Kakehashi basin residents by Li et al. [7] also revealed a higher rate of cardiovascular mortality among female participants. Similarly, a follow-up study conducted in Spain by Domingo-Relloso et al. [34] found that higher urinary cadmium levels were associated with increased cardiovascular risk in the general population. Although the precise mechanism of Cd-induced damage to blood vessels remains unclear, individuals residing in Cd-exposed areas should be mindful of their cardiovascular health.

Our research indicates that higher levels of urinary MT in women are associated with a wider range of diseases leading to mortality compared to men. While the Cox proportional-hazards model found a connection between urinary MT concentration and all-cause mortality in men, women also showed an association with circulatory diseases (cardiovascular diseases) and renal causes of death. Previous studies on Cd-exposed residents in the Jinzu River Basin [25, 35] and Kakehashi River Basin [2, 7, 21] further support these findings, suggesting that women may be more susceptible to the adverse health effects of Cd exposure due to lower levels of iron ions in their bodies [36].

The Ogoya mine, which contaminated the Kakehashi basin, primarily extracted Cu, and it’s possible that other heavy metals besides cadmium were present in the polluted river water. However, according to Nakajima et al. reported [37], only the levels of Cd in urine were higher in the affected area compared to the control area, with no significant differences in Cu and Zn levels. It is reasonable to infer that Cu, being a valuable commodity, would not have been intentionally discarded into the river. Cd, which was not in demand at the time, was likely disposed of in the river, leading to downstream pollution.

As is well known, the development of different countries and regions in the world is uneven. Some developing countries and regions are experiencing the same things that developed countries have experienced. For example, the introduction of polluting and energy-consuming industries eliminated by industrial upgrading in developed countries has caused serious pollution to the environment in the process of economic development. Therefore, people who suffer from Cd pollution may exist for a long time in the future, and some people may be more serious, such as miners and residents in mining cities, workers in metallurgy and electroplating factories, and residents around the plant [38, 39]. This is a serious health hazard for these workers, especially. We suggest that more attention should be paid to urinary MT levels in this population during health examinations. Compared with blood sample collection, urine sample collection is simpler, faster, less costly, and suitable for screening large numbers of people. Our findings provide a reference for early detection and timely prevention of potentially high disease-related mortality risk.

Our research benefits from a lengthy follow-up period spanning from the 1981–1982 health impact survey to November 2016, as well as a sizable sample size, which allowed us to analyze the risk factors for death in Cd-exposed residents of the Kakehashi River Basin. We employed the Cox proportional-hazards model and the Fine and Gray competing risks regression model to consider the risk of death from various diseases and the competitive relationships between these causes of death. Our study found several causes of death associated with higher urinary MT concentrations. However, we must recognize that our study has some limitations. The first is that in 1982, we collected basic information and urine samples from more than 3,000 participants; unfortunately, we did not collect detailed information about the participants’ lifestyle habits, personal medical history, and environmental factors that might influence mortality. Especially regarding smoking, it is well-known as a significant risk factor for lung cancer, COPD, and cardiovascular diseases [40], and it also leads to the intake of certain levels of Cd. Moreover, while alcohol has a more complex relationship with health, infrequent, moderate, and low levels of alcohol consumption are associated with reduced all-cause mortality and cause-specific mortality rates [41]. However, according to findings from various studies, smoking has a minimal influence on the association between Cd exposure and the risk of fractures, breast cancer [42, 43], and all-cause mortality [44]. Additionally, in several studies of the association between cadmium exposure and cardiovascular disease mortality, adjustments were made not only for smoking status, but also for smoking intensity (in pack-years), alcohol consumption, and medication effects, and the significant associations between cadmium exposure and adverse cardiovascular outcomes persisted even after these combined adjustments were made [45, 46]. These differences highlight the need for further research to clarify the exact impact of lifestyle habits on mortality in Cd-exposed areas. Second, although at the beginning of our study, in addition to collecting samples from all the inhabitants of 23 villages with cadmium contamination (rice containing more than 0.4 µg Cd/g of cadmium), 132 men and 159 women were also collected from two villages with no Cd exposure, as we all know, the follow-up of more than 3,000 people is not an easy task. Therefore, during the follow-up period, we focused on the population living in the 23 cadmium-contaminated villages. In addition, for comparison purposes, we divided the MT into four groups according to concentration. However, the control group was established to exclude the influence of other factors on the study’s results, and the absence of a control group may have led to a lack of generalizability of the results.

## 5. Conclusions

In the follow-up study of individuals exposed to Cd in the Kakehashi River Basin in Japan, it was found that elevated urinary MT levels were linked to increased overall mortality, as well as higher cancer-related mortality in men and cardiovascular disease-related mortality in women. Urinary MT levels could potentially serve as an indicator of mortality from these specific diseases in populations chronically exposed to Cd. Furthermore, the study revealed that women with chronic Cd exposure experienced worse health outcomes compared to men, underscoring the importance of focusing on the health status of these women.

## Abbreviations

MT: metallothionein
Cd: cadmium
β_2_-MG: β_2_-microglobulin
RR: relative risk
HR: Hazard Ratio
Cr: creatinine
CI: confidence interval
GSD: Geometric standard deviation
Cu: copper
Zn: zinc

## Supplementary information

**Additional file 1:** **Table S1.** The ratio of MT concentration to the standard deviation of the corresponding sex was subjected to logarithmic processing and the age-adjusted risks of all-cause and cause-specific mortality among all residents. **Table S2.** The ratio of MT concentration to the standard deviation of the corresponding sex was subjected to logarithmic processing and the age-adjusted risks of all-cause and cause-specific mortality among male residents. **Table S3.** The ratio of MT concentration to the standard deviation of the corresponding sex was subjected to logarithmic processing and the age-adjusted risks of all-cause and cause-specific mortality among female residents.

## Declarations

### Availability of data and materials

Data will be made available on request.
