# Supplementary material for: Association between urinary metallothionein concentration and causes of death among cadmium-exposed residents in Japan: a 35-year follow-up study
Source: Environ Health Prev Med. 2025 Jan 7;30:1. doi: 10.1265/ehpm.24-00176 (PMC11744029; doi:10.1265/ehpm.24-00176)
Supplement: Supplementary file 1 — Additional file 1: Table S1. The ratio of MT concentration to the standard deviation of the corresponding sex was subjected to logarithmic processing and the age-adjusted risks of all-cause and cause-specific mortality among all residents. Table S2. The ratio of MT concentration to the standard deviation of the corresponding sex was subjected to logarithmic processing and the age-adjusted risks of all-cause and cause-specific mortality among male residents. Table S3. The ratio of MT concentration to the standard deviation of the corresponding sex was subjected to logarithmic processing and the age-adjusted risks of all-cause and cause-specific mortality among female residents. [file ehpm-30-001-s001.docx]

**Association between urinary MT concentration and causes of death among cadmium-exposed residents in Japan: a 35-year follow-up study**

Lianen Li^1^, Rie Okamoto^2^, Xian Liang Sun^1, 2*^, Teruhiko Kido^2**^, Kazuhiro Nogawa^3^, Yasushi Suwazono^3^, Hideaki Nakagawa^4^, Masaru Sakurai^4^

^1^ School of Medicine, and The First Affiliated Hospital, Huzhou University, 759 2nd Ring East Road, Huzhou 313000, China

^2^ Faculty of Health Sciences, Institute of Medical Pharmaceutical and Health Sciences, Kanazawa University, 5-11-80 Kodatsuno, Kanazawa, Ishikawa, Japan

^3^ Department of Occupational and Environmental Medicine, Graduate School of Medicine, Chiba University, 1-8-1, Inohana, Chuoku, Chiba, Japan

^4^ Department of Epidemiology and Public Health, Kanazawa Medical University, 1-1 Daigaku, Uchinada, Ishikawa, Japan

*Corresponding author: School of Medicine, and The First Affiliated Hospital, Huzhou University, 759 2nd Ring East Road, Huzhou 313000, China

E-mail address: [syh9012881999@163.com](mailto:syh9012881999@163.com) (Xian Liang Sun).

**Corresponding author: Faculty of Health Sciences, Institute of Medical Pharmaceutical and Health Sciences, Kanazawa University, 5-11-80 Kodatsuno, Kanazawa, Ishikawa, Japan

E-mail address: [tkido@staff.kanazawa-u.ac.jp](mailto:tkido@staff.kanazawa-u.ac.jp) (Teruhiko Kido)

Table S1. The ratio of MT concentration to the standard deviation of the corresponding sex was subjected to logarithmic processing and the age-adjusted risks of all-cause and cause-specific mortality among all residents.

| Cause of death | N | HR per SD (95%CI) | P |
| --- | --- | --- | --- |
| All-cause of death | 2218 | 0.90 (0.81-1.00) | 0.06 |
| Cause-specific death |  |  |  |
| Malignant neoplasms | 543 | **0.68 (0.54-0.85)** | **＜0.001** |
| Endocrine, nutritional, and metabolic diseases | 40 | 1.28 (0.57-2.88) | 0.56 |
| Circulatory diseases | 717 | 1.00 (0.83-1.20) | 0.97 |
| Cardiovascular diseases | 367 | 0.94 (0.73-1.22) | 0.66 |
| Cerebrovascular diseases | 322 | 1.13 (0.86-1.49) | 0.39 |
| Diseases of the respiratory systems | 327 | **0.71 (0.54-0.95)** | **0.02** |
| Diseases of the digestive systems | 97 | 1.37 (0.83-2.27) | 0.21 |
| Kidney and urinal tract diseases | 75 | **1.72 (1.00-2.97)** | **0.05** |
| Senility | 172 | 0.98 (0.68-1.41) | 0.91 |
| External causes of mortality | 104 | 1.06 (0.64-1.76) | 0.82 |

MT: metallothionein, HR: hazard ratio, CI: confidence interval.

Bold figures show significant HR.

Table S2. The ratio of MT concentration to the standard deviation of the corresponding sex was subjected to logarithmic processing and the age-adjusted risks of all-cause and cause-specific mortality among male residents.

| Cause of death | N | HR per SD (95%CI) | P |
| --- | --- | --- | --- |
| All-cause of death | 1104 | **1.21 (1.03-1.42)** | **0.02** |
| Cause-specific death |  |  |  |
| Malignant neoplasms | 311 | 1.00 (0.73-1.37) | 1.00 |
| Endocrine, nutritional, and metabolic diseases | 15 | 1.24 (0.29-5.38) | 0.77 |
| Circulatory diseases | 325 | 1.24 (0.93-1.67) | 0.15 |
| Cardiovascular diseases | 160 | 1.10 (0.72-1.68) | 0.66 |
| Cerebrovascular diseases | 146 | 1.48 (0.96-2.29) | 0.08 |
| Diseases of the respiratory systems | 188 | 1.26 (0.85-1.86) | 0.24 |
| Diseases of the digestive systems | 46 | **2.40 (1.11-5.21)** | **0.03** |
| Kidney and urinal tract diseases | 38 | 2.16 (0.96-4.89) | 0.06 |
| Senility | 54 | 0.76 (0.37-1.55) | 0.45 |
| External causes of mortality | 58 | 1.61 (0.79-3.29) | 0.19 |

MT: metallothionein, HR: hazard ratio, CI: confidence interval.

Bold figures show significant HR.

Table S3. The ratio of MT concentration to the standard deviation of the corresponding sex was subjected to logarithmic processing and the age-adjusted risks of all-cause and cause-specific mortality among female residents.

| Cause of death | N | HR per SD (95%CI) | P |
| --- | --- | --- | --- |
| All-cause of death | 1114 | **1.22 (1.04-1.42)** | **0.01** |
| Cause-specific death |  |  |  |
| Malignant neoplasms | 232 | 1.06 (0.74-1.53) | 0.75 |
| Endocrine, nutritional, and metabolic diseases | 25 | 1.49 (0.51-4.37) | 0.47 |
| Circulatory diseases | 392 | 1.23 (0.95-1.59) | 0.12 |
| Cardiovascular diseases | 207 | 1.15 (0.81-1.63) | 0.44 |
| Cerebrovascular diseases | 176 | 1.35 (0.92-1.99) | 0.13 |
| Diseases of the respiratory systems | 139 | 1.02 (0.66-1.58) | 0.93 |
| Diseases of the digestive systems | 51 | 1.40 (0.70-2.80) | 0.35 |
| Kidney and urinal tract diseases | 37 | **2.94 (1.38-6.23)** | **0.01** |
| Senility | 118 | 1.07 (0.68-1.68) | 0.77 |
| External causes of mortality | 46 | 1.54 (0.69-3.41) | 0.29 |

MT: metallothionein, HR: hazard ratio, CI: confidence interval.

Bold figures show significant HR.
